# Provings and Clinical Observations with High Potencies

**Published:** 1893-10

**Authors:** Malcolm Macfarlan

**Affiliations:** Philadelphia, Pa.


### PROVINGS AND CLINICAL OBSERVATIONS WITH HIGH POTENCIES.

MALCOLM MACFARLAN, M. D., PHILADELPHIA, PA.

### SASSAFRAS$^{5C}$.

Severe strangury, urine looks as if it had portions of mucus and blood mixed with it.

### STRAMONIUM$^{CM}$.

Urine the *consistence of oil;* scanty; *very brown* color, like old ale, thick from mucus and excess of phosphates.

### SULPHUR$^{CM}$.

His urine soon became turbid, like yeast.

Unable to pass urine. Compelled to use a catheter.

### STRONTIA$^{5C}$.

Great strangury. Frequent and profuse urination.

### TEREBINTH.$^{17M}$.

Caused urination every ten minutes, symptom lasting several hours.

Often cured gonorrhœa with it.

Trouble to get his urine to flow for five minutes; one prover fourth day after taking medicine.

Cured a girl, aged five, who since birth had but little control over the functions of urine or bowels.

THERIDION.$^{5C}$.

Water scanty, high-colored, constipation.

After going to bed has to get up four or five times to pass water; does not secrete much in daytime.

UVA-URSA$^{10M}$.

Cutting sensation on urination—burning soreness over the whole body, as if bruised.

VALERIAN$^{CM}$.

Increased his flow of urine to twice its usual quantity.

ZINC-SULPH.$^{5C}$.

Compelled to get up two or three times in the night to urinate.

### GENITALS.

AGARIC.$^{2M}$.

Cramps, as if she was going to have a child; violent bearing-down pain. When she trod on the floor she could cry out, it hurt her so in the right ovarian region.

Eruption on genitals like little hard pimples.

ARUM-TRI.$^{16M}$.

Caused smarting at end of penis.

COPAIBA$^{CM}$.

Given in water every two hours produced symptoms of strangury, like gonorrhœa.

KALI-HYD.$^{CM}$.

Cured scabby, itching eruption in labia of a young girl.

Often verified this symptom in curing itching of genitals in women.

### MEN.

### Cinnabar.

Spots on the glans penis like herpes.

### Carb-veg.$^{76M}$.

Produced watery discharge from urethra.

### Cadmium$^{5C}$.

Sulphate of Cadmium and other preparations of it remarkably efficacious in checking the too frequent nightly seminal emissions of young men ; often verified.

### Clematis$^{5C}$.

Has cured many cases of swelled testicle of long standing, often attending with great pain and faintness.

### Digitalis$^{CM}$.

Produced severe urethritis, phymosis, and strangury.

Symptoms appeared on fourth day in a case of hydrothorax. Medicine given every half-hour.

Have cured very many cases of gonorrhœa with this remedy.

### Gettysburg-salts$^{45M}$.

*Emissions.* Blood with them.

### Mer-corr.$^{5C}$.

Left testicle swollen.

### Physostigma$^{CM}$.

Calabar bean produced swelling of testicle (lacks confirmation).

### Sulphur$^{CM}$.

Given every two hours for a week, caused three or four seminal emissions daily.

### WOMEN.

### AGARIC.$^{2M}$.

Cured completely a bearing-down sensation. I had been treating her for one and a half years without effect. The pain came on again (when without medicine), but eventually cured.

Cured a chronic bearing-down pain lasting years; verified often; soreness in abdomen over the ovaries.

A most reliable remedy in ovarian soreness, left side being mostly affected.

### ARUM-TRI.$^{16M}$.

Her menses, which had been absent two months, returned. Pain in left mammæ as if bruised.

### BURSA-PAST.$^{9M}$.

The girl had menstruated the week before scantily; it came on again very profusely; she had previously had amenorrhœa.

Useful in menstrual irregularities of young women.

### CAUST.$^{5C}$.

Thick yellow discharge from vagina.

### CIMICIFUGA-RAC.$^{95M}$.

Menses usually very regular, delayed three weeks.

In a pregnant woman relieved pain in abdomen and uterus, with severe bearing-down sensation; sympathetic pain around the heart.

Cure was rapid and complete—verified many times.

### BELLADONNA$^{CM}$.

Has acted in a miraculous manner in curing hard swelling of the glans; principally in breasts of women, existing in one case six years, in the other one. Useful in glandular enlargements of the neck.

### ERIGERON$^{45M}$.

Checked uterine hemorrhages, amenorrhœa when Ferrum$^{CM}$. did no good. Turpentine acted as well as Erigeron in three cases.

### HELLEBORE$^{CM}$.

Cured several cases of amenorrhœa in young girls.

### JASPER$^{15C}$.

Leucorrhœa of mild, persistent type.

### KALI-CARB.$^{24M}$.

Rectified insufficient labor pains with violent pain in *the small of the back.*

### MYRRH$^{5C}$.

Cured indescribably severe uterine pains of a transient nature.

### NITRUM$^{5C}$.

Cured cramp-like pains during menstruation, had for years this peculiar pain during menses. Was completely cured.

### OXALIS$^{1M}$.

Dragging sensation from hip to groin in ovarian region, feels as if everything would be pressed out, urinates frequently; pain from her kidney to her hip causes her to be so bent she could scarcely straighten up.

### PHOSPHORUS$^{2C}$.

Cured a distressed sensation in left ovary, soreness to touch. Prover a consumptive—symptom not prominent.

### POLYGONUM$^{42M}$.

Menses returned, but were scanty and very dark. Case under treatment for amenorrhœa.

### SEPIA$^{45M}$.

Brought back the menses at once in a young woman who had amenorrhœa six months, another case eight months, both pale, weak, thin, sickly. Verified in many other cases.

*Sepia* is the best and most active general remedy for prolapsus uteri, backache, ovarian pain, and leucorrhœa in elderly women.

Cured a woman, æt. fifty, with pains in left groin, severe backache, bearing-down sensation, uterus often making its appearance outside. Urination difficult and painful. Troubled twenty years. Complete relief.

### SENEGA$^{5C}$.

Menses came on too soon.

### SILICEA$^{CM}$.

Helps greatly, dragging sensation in lower abdomen occurring with women. Relieved pain in left ovary.

### SYMPHYTUM$^{15C}$.

Menses cease for a month when proving.

### STRONTIA-CARB.$^{3M}$.

Show of menses all the time since last period.

### TEREBINTH$^{17M}$.

Cured a woman, æt. forty, an invalid for many years, with a violent burning sensation in the uterus, bearing-down pain. Caused a feeling of great heat over the body, now craves liquids.

Cured a remarkable case of hemorrhage from uterus, woman æt. fifty, bleeding for two years every day; checked in one week; causes in provers watery-like discharges from the uterus.

### VERBASCUM$^{45M}$.

Menses come on earlier than usual.

### CHILDREN.

### APIS$^{CM}$.

Helped children teething in summer, particularly with symptoms of cerebral congestion; loose bowels; no vomiting. Repeated verifications of this. Not so generally useful as Creosote.

BELLAD.[101M].

Has nearly always promptly helped children with hip-joint disease, as to the acute pain and otherwise.

CAMPHOR[CM].

Promptly cured many times the watery diarrhœa of children, often attended with colic and vomiting.

CALC-CARB.[97M].

Cured a child aged one and a half years, wasted with bronchitis; no diarrhœa, no vomiting; where Tar., Lyc., Sulph. did no good.

CINA[12C].

Caused violent frontal headache, nervousness, twitching, fever, and sweat.

